# SingleScan: a comprehensive resource for single-cell sequencing data processing and mining

**DOI:** 10.1186/s12859-023-05590-9

**Published:** 2023-12-07

**Authors:** Kun Wang, Xiao Zhang, Hansen Cheng, Wenhao Ma, Guangchao Bao, Liting Dong, Yixiong Gou, Jian Yang, Haoyang Cai

**Affiliations:** 1 Center of Growth, Metabolism and Aging, Key Laboratory of Bio-Resource and Eco-Environment of Ministry of Education, College of Life Sciences, Sichuan University, Chengdu, 610065 China; 2 Department of Breast Surgery, Sichuan Provincial People’s Hospital, University of Electronic Science and Technology of China, Chengdu, 611731 China; 3 Chinese Academy of Sciences Sichuan Translational Medicine Research Hospital, Chengdu, 610072 China

**Keywords:** Single cell sequencing, Data processing pipeline, Tools development, Single-cell transcriptome, Benchmark

## Abstract

Single-cell sequencing has shed light on previously inaccessible biological questions from different fields of research, including organism development, immune function, and disease progression. The number of single-cell-based studies increased dramatically over the past decade. Several new methods and tools have been continuously developed, making it extremely tricky to navigate this research landscape and develop an up-to-date workflow to analyze single-cell sequencing data, particularly for researchers seeking to enter this field without computational experience. Moreover, choosing appropriate tools and optimal parameters to meet the demands of researchers represents a major challenge in processing single-cell sequencing data. However, a specific resource for easy access to detailed information on single-cell sequencing methods and data processing pipelines is still lacking. In the present study, an online resource called SingleScan was developed to curate all up-to-date single-cell transcriptome/genome analyzing tools and pipelines. All the available tools were categorized according to their main tasks, and several typical workflows for single-cell data analysis were summarized. In addition, spatial transcriptomics, which is a breakthrough molecular analysis method that enables researchers to measure all gene activity in tissue samples and map the site of activity, was included along with a portion of single-cell and spatial analysis solutions. For each processing step, the available tools and specific parameters used in published articles are provided and how these parameters affect the results is shown in the resource. All information used in the resource was manually extracted from related literature. An interactive website was designed for data retrieval, visualization, and download. By analyzing the included tools and literature, users can gain insights into the trends of single-cell studies and easily grasp the specific usage of a specific tool. SingleScan will facilitate the analysis of single-cell sequencing data and promote the development of new tools to meet the growing and diverse needs of the research community. The SingleScan database is publicly accessible via the website at http://cailab.labshare.cn/SingleScan.

**Supplementary Information:**

The online version contains supplementary material available at 10.1186/s12859-023-05590-9.

## Introduction

Single-cell sequencing comprises a suite of technologies and approaches that interrogate the sequence or chromatin information at the single-cell level. At present, single-cell sequencing is widely used in many cutting-edge biological research fields. In recent years, further advancements in the form of single-cell ChIP-seq [1–6], ATAC-seq [7, 8], and spatial transcriptomics technologies continued to emerge [9]. The popularity of these techniques has increased their robustness and made them available to more biological researchers [10]. Recently, single-cell sequencing was used to identify and profile immune response in patients with coronavirus disease 2019 (COVID-19) [11].

As advances in experimental technology have motivated large-scale innovation in computational methods [12], a number of bioinformatics tools and software have become available for the analysis of single cell sequencing data. The availability of computational frameworks and software repositories such as Bioconductor [13], Seurat [14–18] and Scanpy [19], has allowed researchers to navigate this space and build analysis pipelines. Further, several resources have been established for curating and integrating single-cell sequencing data. For instance, CancerSEA [20], scRNASeqDB [21], and PanglaoDB [22]collected public data on single-cell researches and created integrated analysis database. These databases focus on data collection, annotation, and visualization. scRNA-tools [23, 24] is a tool database which collects the information of single-cell RNA sequencing-related tools.

However, a primary unsolved challenge in this field is to select appropriate tools from many alternatives to build optimal data processing pipelines. Another daunting but important task is choosing suitable parameters for each tool, particularly for researchers without bioinformatics expertise. Thus, a resource devoted to providing easy access to detailed information on single-cell sequencing methods and single-cell sequencing data processing pipelines is urgently needed.

With the development of technology, the analysis process has become more complex. Lukas et al. review a single-cell (multi-)omics analysis and guides advanced users to the most recent best practices [12], making it possible for us to summarize a single-cell analysis workflow to suggest comprehensive practice workflow for the most common analysis steps. In the present study, SingleScan, a manually curated resource for single-cell transcriptome/genome analysis pipeline and usage scenarios, was developed. At present, > 1500 tools and 300 publications have been integrated in this resource. SingleScan enables users to quickly explore the features of each tool and role of the tool in the entire data analysis procedure. Meanwhile, SingleScan builds a benchmark pool that collects the published benchmark articles that it produces the best practices recommendations for approaching a standard analysis. Thus, it facilitates users to select and integrate appropriate tools into their own data processing pipelines. Furthermore, SingleScan includes the classic single-cell analysis methods and related source code links, enabling the users to easily initiate their analysis. The statistics based on all the curated tools will help researchers track recent trends in single-cell based studies and methods development. As SingleScan curates almost all the tools that have been developed so far, it presents the state of the art for data analysis in the single-cell sequencing technology.

In general, SingleScan provides a relatively comprehensive list of single-cell analysis tools and provides a standard process for single cell analysis, with software available for each step. The single-cell research literature integrated in the database includes multi-omics sequencing technologies [25] such as CITE-seq [26] and scTrio-seq [27]. Rather than being limited to only one technology, some studies have examined two or more omics simultaneously, such as the combined analysis of the scRNA-seq and scATAC-seq [7, 8]. Users can learn about the methods used in the analysis of these multi-omics articles. In addition, the species covered include human, mouse and other model species. It also integrates published benchmark articles to recommend tools based on specific single-cell analysis methods such as quantification and clustering.

## Methods

### Data collection

To retrieve all related publications, we first used a Python program to get thousands of DOI numbers of publications on PubMed using the following set of keywords: "single cell sequencing", "single-cell tool", "single cell analysis", "single-cell benchmark", and "scRNA-seq". Then we saved them in our local single-cell publication library (scLibrary). Next, we manually searched on PubMed to view the detailed information of the article through the DOI number and selected appropriate articles to add to SingleScan. An article was eligible for inclusion if it met at least one of the following criteria: (1) the study designed a tool for single-cell data analysis or contained such a module; (2) the study provided a specific tool for users to download or use online; (3) the tool was open source and free for noncommercial academic use; and (4) the study included data processing at the single-cell level; (5) they performs benchmark studies on single cell analysis methods. In total, 300 more representative publications that studied multiple model species were collected based on a standard scRNA-seq analysis used in the publication and the species studied including human, mouse, zebrafish, Arabidopsis, maize, and western claw-toed frog. In addition, articles containing 1587 tools were used for single-cell analysis and 40 benchmarking publications were collected for the subsequent data curation process. The Python program code is available up on GitHub (https://github.com/victorwang123/SingleScan).

### Data processing

The main text and additional files of each publication were carefully examined, and the single-cell data analysis tools and their specific parameters used in these studies were extracted. Other meaningful information, including sequencing platform, disease type, number of sequenced cells, and patients’ clinical data, was also collected, subject to availability. Such information was organized at both publication and tool levels. The basic information of tools, including the platforms used to build the tools, links to code repositories, and short descriptions, was extracted from GitHub, Bioconductor, The Comprehensive R Archive Network, and The Python Package Index. The usage code was extracted from its documentation. For each tool, the citations of the article since it published was collected using the Python program. Also, we added the citations in the past year and calculated the average annual citations. To facilitate users for choosing appropriate tools, an overall evaluation score (x'), which employed min–max scaling to normalize citations (x), was calculated: 

$${x}^{\prime}=\frac{x-min\left(x\right)}{max\left(x\right)-min\left(x\right)}$$

Tools were marked using different colors and can be sorted according to the evaluation score. This score is a scale of the citations of all tools so that it is in the range of 0–10, so we assume that the higher the score, the higher the citations of that tool.

### Data assignment

According to the description in reviews [12, 28] and the research publications we collected, a standard single cell analysis process which was consists of several tasks. Finally, we got a total of 20 functional modules. The literature was analyzed to extract the description of each tool, and has been described in reviews, all the tools were categorized into these 20 functional modules. The description information of each function module has been uploaded in Additional file 1: Table S1. Each tool is categorized according to the analysis tasks it can perform. For each tool, the descriptions in the accompanying paper or document are first checked very carefully, and then a precise "yes" or "no" determination is made manually for each functional module.

### Web interface

The web interface of SingleScan was implemented using HTML, Golang, and JavaScript, with MongoDB used for data storage. The main functional pages include "Search", "Browse", "Benchmark", "Statistics", and "Download". A total of three options are provided in the "Search" page. In the first option Search by Publications, users can obtain detailed information on software, R packages, and parameters that were used in a certain publication. Wherever available, the application scenarios of tools, including the number of cells sequenced, sequencing platform, and clinical information, are also provided. In the second option Search by Tools, users can query for tools using keywords (e.g., clustering, quality control, and others). Finally, in the third option Search by Functions, as all the tools are classified into 20 functional modules, users can search for appropriate tools according to their analysis purposes. In the "Browse" page, users can access tools by clicking the summarized single-cell data analysis pipeline. For each step, users are provided with a list of available tools. Specific details of recommended tools will be available on our "Benchmark" page. Users can query recommended tools for a certain step in the single cell analysis process.

The "Statistics" page presents various statistics based on the collected data. This information will help researchers obtain insights into the current development trends of single-cell level research and gain a quick overview of the specifics of each tool. The "Download" page enables users to access the full data of SingleScan that are organized as per publications, samples, and tools.

### Statistics and data visualization

Python (v3.8.4) and R (v4.0.3) programming languages were used for statistical computing. Data presentation and visualization were performed using Highcharts (v8.8.2), Jsplump (v1.7.10), and G2 (v4.0).

## Results

### Overview of SingleScan

**Fig. 1:**
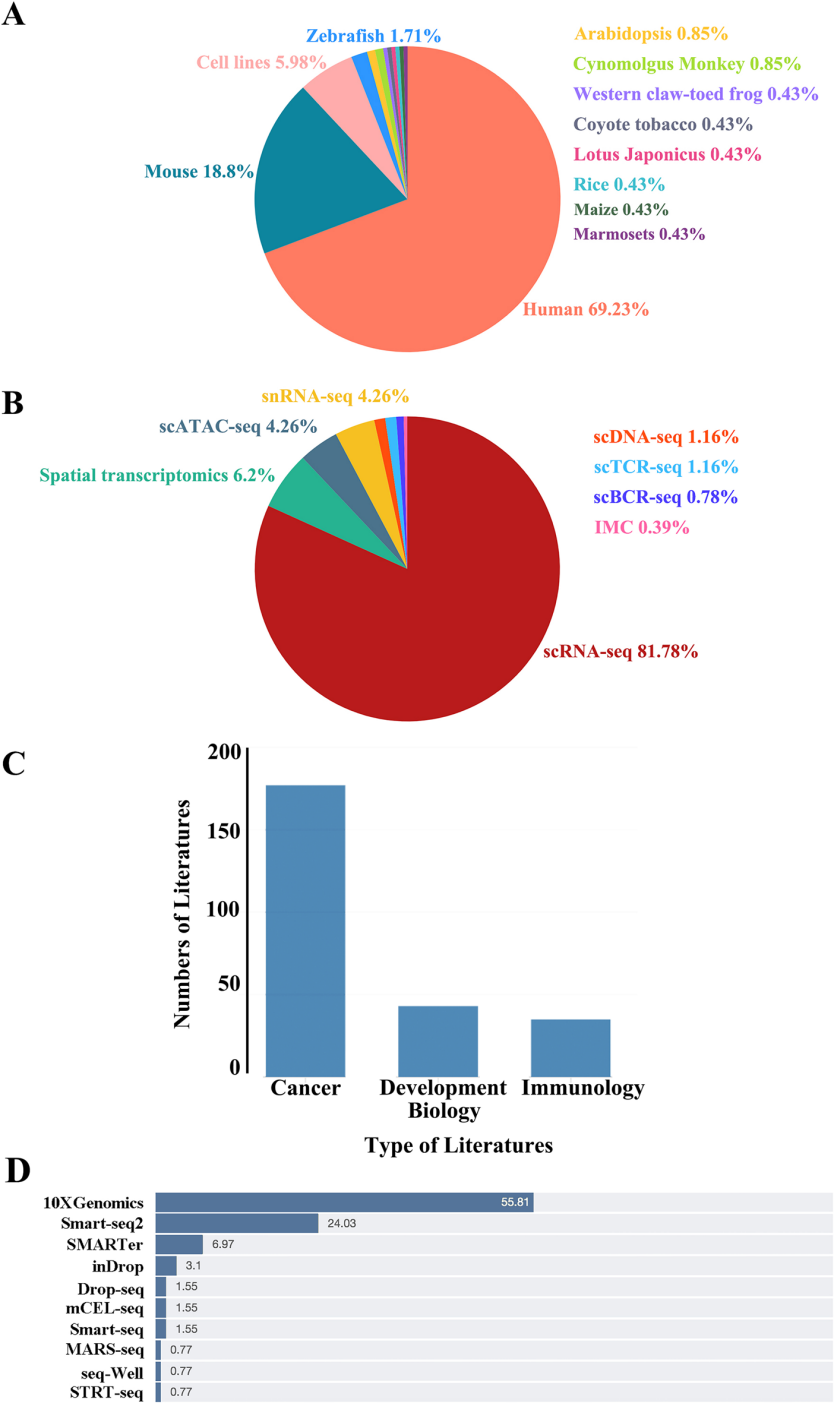
Data content of SingleScan. **A** Pie chart shows the distribution of studies in SingleScan according to related species. **B** Pie chart shows the distribution of single-cell sequencing technologies used in publications. **C** Main research fields of publications that were integrated into SingleScan. **D** Distribution of different single-cell sequencing platforms used in the publications

SingleScan catalogs pipelines and tools for both single-cell transcriptome and genome data analysis, integrating information from 300 research publications that studied several model species, including human, mouse, zebrafish, Arabidopsis, maize, and western claw-toed frog and 1587 method articles used for single-cell analysis (Fig. 1A). In the present study, for data selection, oncology research was considered. It should be noted that in SingleScan, most included studies employed scRNA-seq for creating a transcriptomic atlas (Fig. 1B) and that the main research fields were tumor biology, developmental biology, and immunology (Fig. 1C). Most of the included studies had cell numbers > 30,000. Among them, tumor-related studies accounted for the largest proportion (66%). As for technology platforms, most studies were based on 10X Genomics and Smart-seq2, accounting for 55% and 24% of the total number of studies (Fig. 1D), respectively.

**Fig. 2:**
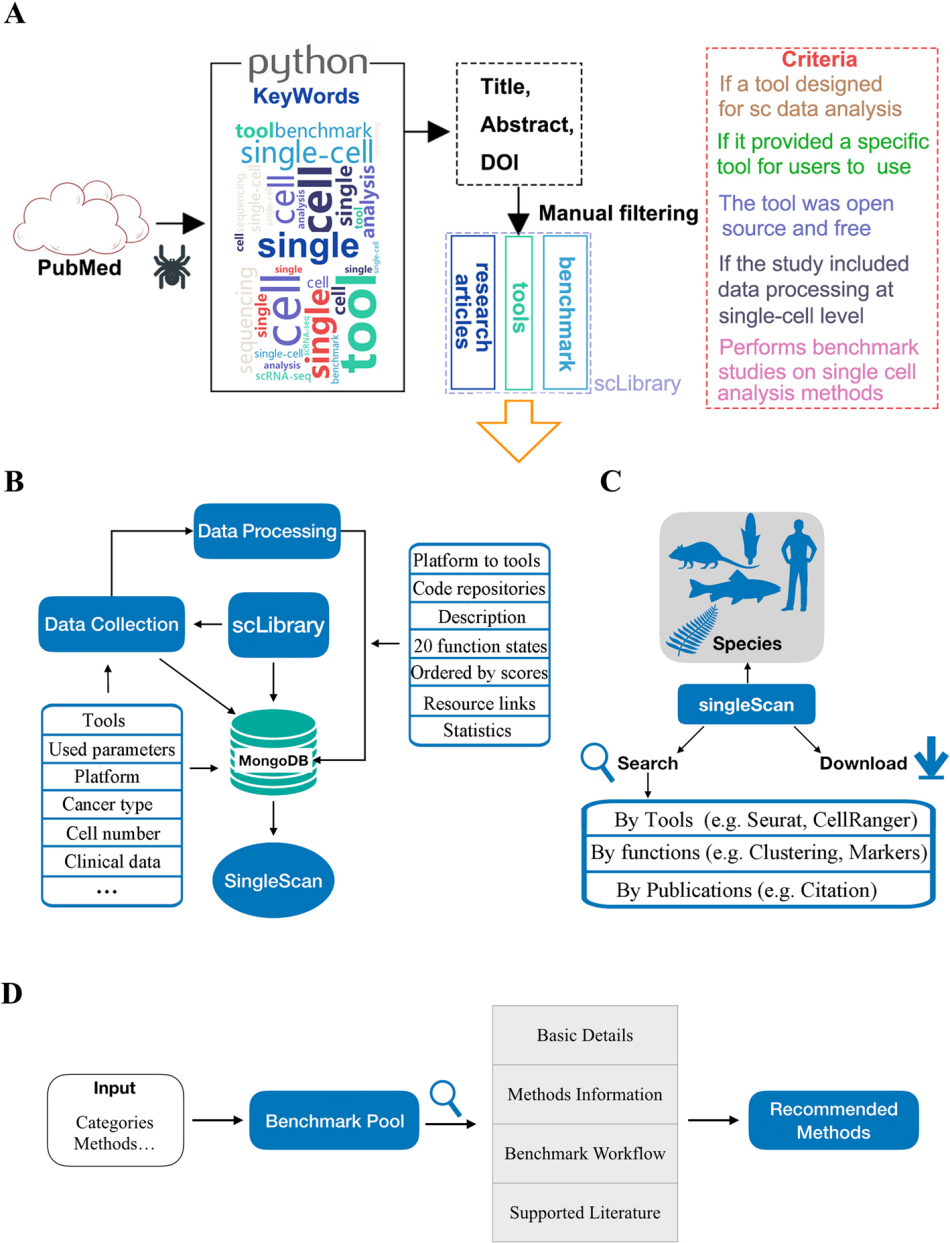
Overview of SingleScan Model. **A** Data collection methods. Using a Python program to get thousands of DOI numbers of publications and return results local single-cell publication library (scLibrary). The tools/studies were then manually curated. **B** The flow chart of data collection and annotation. **C** The main functions of SingleScan include: (1) Search by tools, a feature that allows users to search for tools by functions or related key words (e.g. clustering, quality control). (2) Search by functions, a feature that allows users to search for appropriate tools according to their analysis tasks. (3) Search by publications, a feature that allows users to obtain detailed information about the tools, R packages and parameters that used in each publication, and other related data, including the number of cells, sequencing platforms, clinical information. (4) Data download. **D** SingleScan collects literature on benchmarks for single-cell analysis methods to help users choose the most suitable tool at each step

The workflow of SingleScan construction is shown in Fig. 2. In short, through a Python program, publications that may be relevant to the contents of SingleScan are collected and then process them manually (Fig. 2A). A set of information on each single-cell data analysis tool was collected, and all tools were classified into groups according to their functions (Fig. 2B). The tools were then integrated into a single-cell analysis workflow, which clearly illustrated the function of each step. Users can search these tools via the three search modes (Fig. 2C). Furthermore, a benchmark pool, which contains benchmark studies for each step of single-cell sequencing data analysis, was constructed to provide the list of most suitable methods for a specific purpose (Fig. 2D).

For beginners in the field of single-cell sequencing, SingleScan is useful to quickly get an overview of workflow tasks or track recent trends in methods development. As the parameters and application scenarios from published articles were included, our resource can provide researchers with sufficient information to choose the appropriate tools and optimal parameters. In-house scripts were developed to help automatically parse and obtain the latest usage information of each tool, including links to code, citations, and date of update. This function ensures that the information in our resource is regularly updated.

If a beginner gets a raw data, the first step is to check the process on "Browse" page, and then click this step, the tools that can be used in this analysis step will listed. Users can choose based on the number of citations, or on the "Benchmark" page, check out the recommended tools for this step of the process. Also, users can view the analysis methods and parameters used by other researchers studying the similar area on the "By paper" page (Additional file 1: Figs. S1, S2).

### Analysis workflow of single-cell sequencing data

**Fig. 3:**
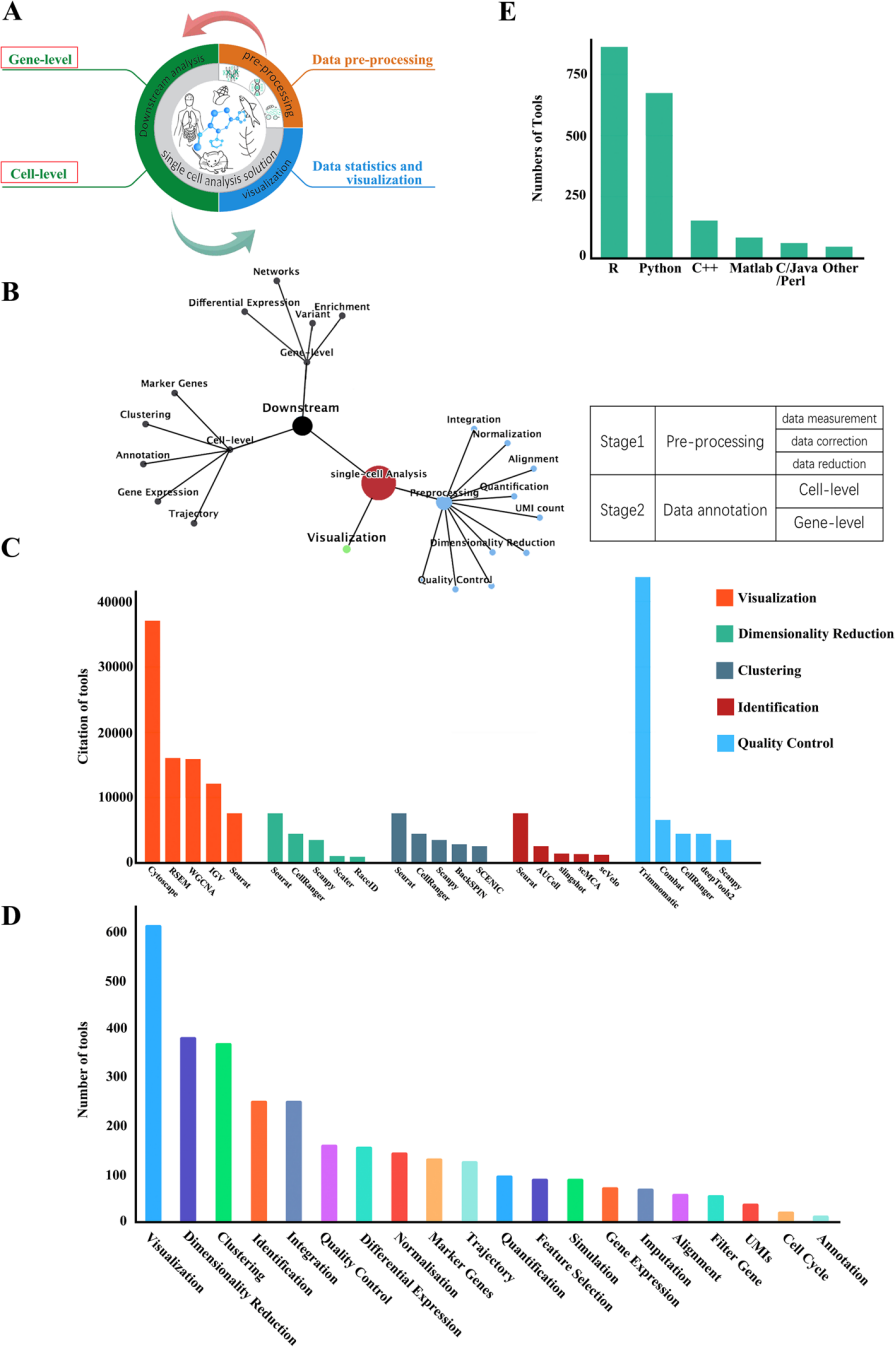
Usage and statistics of SingleScan. **A** Single-cell analysis solution. SingleScan presents a classical single cell analysis pipeline. (1) Pre-processing, where users can choose appropriate tools for data processing (e.g. QC, Alignment, etc.). (2) Downstream analysis, where SingleScan divides this phase into two levels (cell- and gene- level). 3) Data visualization. **B** Single-cell analysis Atlas. The relationship of 20 functional modules. **C** The tools in SingleScan are sorted by citations separately within each functional group. **D** The number of functional modules of different tools in SingleScan. **E** The majority of tools were developed by R or Python programming languages

As novel tools continue to be developed, there are many tools available for each step of single-cell sequencing data analysis. In general, various combinations of tools can be utilized for data analysis. The common analysis workflows were summarized by collating and comparing a large number of related studies. According to their tasks, tools were organized into 20 functional modules. A typical model of single-cell data analysis was summarized and a list of available tools for each step was provided. The data processing workflow can be roughly divided into two stages: preprocessing (including quality control, normalization, data correction, feature selection, and dimensionality reduction) and data annotation (cell and gene levels). The raw data generated from single-cell sequencing platforms are initially processed in Stage 1 (preprocessing). During this stage, raw data are processed via a series of filtering and normalization steps, including reads quality control (QC), assignment of reads to cellular barcodes, and reference genome/transcriptome alignment and quantification. These steps remove potential low-quality reads, eliminate batch effects of gene expression, and transform the raw data into a format that facilitates subsequent analysis. To outline the workflow, this stage was delineated into the following three layers based on the work of Luecken and Fabian [28]: data measurement, data correction, and data reduction. It should be noted that some of the analysis tasks in the preprocessing stage are common to bulk sequencing data analysis, including quality control, normalization, feature selection, and quantification. The clean reads or counts matrices are then passed to Stage 2 (data annotation), which focuses on the extraction of biological insights and elucidation of the underlying biological system. The data annotation stage was further delineated into two layers: cell level and gene level (Fig. 3A). Cell-level annotation typically focuses on distinguishing cell groups and involves the clustering of cells or traces the trajectory from one cell type to another. The highly informative genes can be identified using the gene-level analysis, which includes the marker genes of different cell groups, differentially expressed genes, and genes participating in regulatory networks. The relationship between these modules is shown in Fig. 3B; researchers need to consider relationships between modules when analyzing data. During the analysis, some integration and analyses of the collected data were performed (Fig. 3C, D, Additional file 1: Fig. S3). Using statistics, researchers can count the programming language used by the tools in these steps (Fig. 3E).

### Benchmark of methods for analysis

**Fig. 4:**
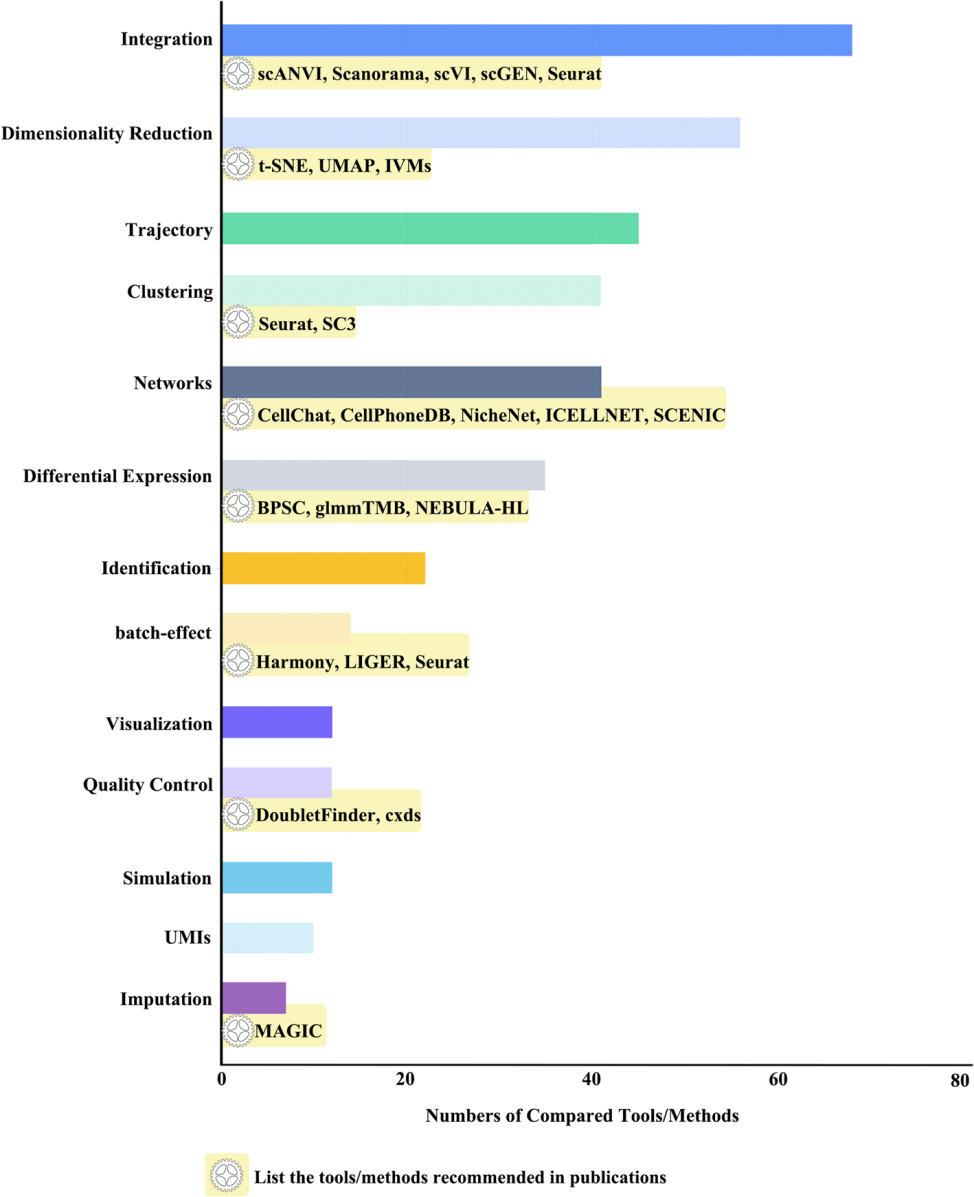
Number of tools compared tools in each tool category

Appropriate methods can enable effective data preprocessing and downstream analyses. As mentioned above, there are many methods for each analysis step. Valuable information can help researchers choose the most suitable methods. However, despite the critical importance of evaluating the effectiveness of methods in the same category, few comprehensive repositories are focused on collecting related information. SingleScan specifically collects literature on the benchmark of these methods and also organizes and categorizes them to build a benchmark pool. There are 15 categories in the benchmark pool of SingleScan, including batch-effect correction, dimensionality reduction, clustering, trajectory reconstruction, differential expression, and others. More than 10 methods were comprehensively compared for each category; such information provides important guidelines for choosing appropriate methods for analysis (Figs. 4, Additional file 1: Fig. S4).

Despite different single cell analysis methods may have different merits for different tasks, and it is not straightforward to identify a single method that strives the best in all data sets and for all downstream analyses, we hope that our database can provide a relatively comprehensive practical guideline for choosing methods in scRNA-seq analysis. There will be specific details of recommended tools in benchmark section. For example, users can search for "dimensionality reduction", a total of 18 tools were compared. In addition to the specific information of each tool, SingleScan also collected their datasets, processes, and which scenarios are suitable for which tool information (Additional file 1: Fig. S4).

### Research hotspots

**Fig. 5:**
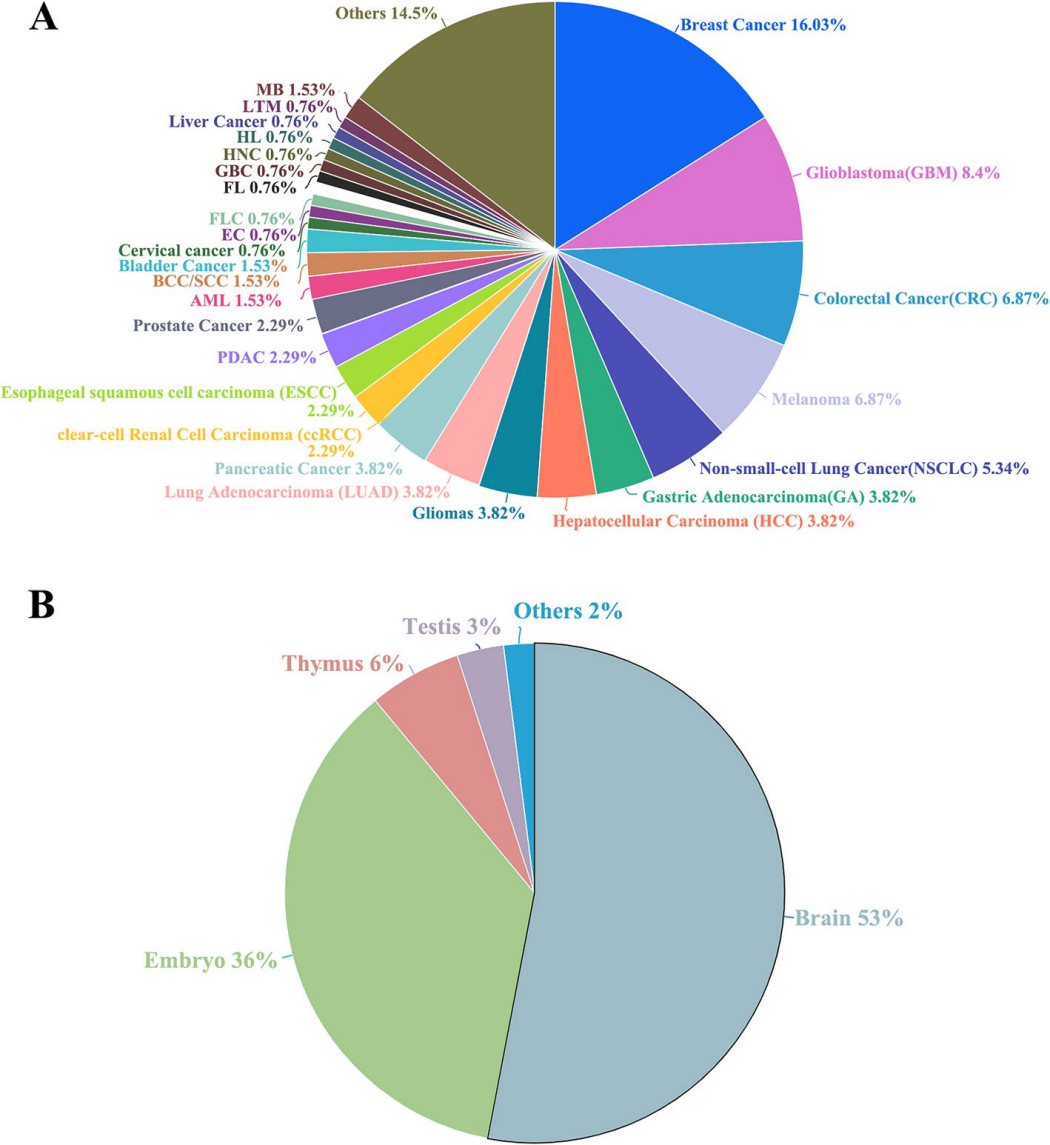
The distributions of cancer type and tissue type in SingleScan. **A** Pie chart shows the percentage of cancer types in SingleScan. **B** The percentage of different tissues or organs involved in studies collected in SingleScan

According to the collected data, many studies based on single-cell sequencing primarily focused on the understanding of mechanisms that underlie tumor heterogeneity. The high-throughput capacity and high resolution of single-cell sequencing have greatly improved the ability to perform specific profiling of cell populations and decipher the functional heterogeneity of cancer cells. With the widespread application of this technology, many significant new insights into cancer development, evolution, and tumor microenvironment have been revealed. SingleScan includes > 300 cancer-related publications containing 49 cancer types. Breast cancer research accounts 14% of the included studies (Fig. 5A). The two other main research areas include developmental biology and immunology. The main objectives of immunology-related studies were to detect changes in immune cell gene expression under various disease states and induction conditions as well as to identify immune cell marker genes and trajectories in different directions of differentiation. The tissue types involved in developmental biology research were primarily the brain and embryo, accounting for 53% and 36% of the total number of studies (Fig. 5B), respectively.

Recently, a novel coronavirus (CoV), designated severe acute respiratory syndrome (SARS)-CoV-2, led to the COVID-19 pandemic, which rapidly spread globally and has been proclaimed a severe public health emergency of international concern by the World Health Organization. Thus, several publications on the single-cell analysis of SARS-CoV-2 were integrated in the SingleScan database. The studies focused on revealing immune system response in patients with COVID-19 (Additional file 1: Fig. S5). These publications have more in-depth research on COVID-19 and have made major breakthroughs in the development of vaccines and response of vaccinators.

Most studies included in the SingleScan resource employed scRNA-seq for creating a transcriptomic atlas of every cell type in a sample (Fig. 1B). Recent publications suggest that the number of cells sequenced in a single study is growing dramatically and that multi-omics analysis at the single-cell level is also increasing. Single-cell sequencing could therefore become a routine tool in biological and biomedical research in the future.

### Trends in methods development

All the curated tools were categorized into 20 functional modules, and statistical analysis was performed on each module. With respect to the programming languages, developers used various languages to build data processing tools. The most popular one was R, followed by Python and C++ (Fig. 3E). The choice of the programming language determines the execution environment of the tool, although some tools support cross-environment processing. Both R and Python are among the most popular programming languages in the field of data mining, which partly explains why they are the most commonly used languages for tool development. As the demand for data analysis continues to increase, more and more tools can possess two or more functional modules. Tools that provide integrated environment for developers and contain analysis toolboxes, such as Seurat [14–18], Monocle [29–31], and Scanpy [19, 32], are more popular. For the analysis steps shared by both bulk and single-cell sequencing, pipeline developers tend to utilize existing tools for bulk sequencing, including BWA [33], edgeR [34], and Bowtie2 [35]. Among all the functional modules, the number of tools that perform data visualization is the largest, followed by clustering, which enables researchers to infer the identity of member cells, with the second largest number of tools. This function is one of the specific and most important advantages of the single-cell sequencing technology. The use of sequencing platforms is closely related to the popularity of certain tools. For example, with the widespread use of the 10X Genomics platform, the usage frequency of CellRanger [36], which is used for analyzing raw data generated using 10X Genomics, has increased dramatically. With the extensive application of single-cell sequencing, more automated and interactive data analysis toolboxes or pipelines are expected to be developed, particularly for some important analysis steps, including clustering and trajectory inference.

## Discussion

SingleScan is a comprehensive resource that curates single-cell transcriptome/genome analysis pipelines and related information. It is aimed to meet the growing demand from the scientific community to manage the ever-increasing number of bioinformatic tools. There are several features that distinguish SingleScan from other similar resources. First, to the best of our knowledge, SingleScan collects a relatively comprehensive list of single-cell sequencing data analysis tools and a portion of the currently available tools for single-cell and spatial transcriptomics solutions (Fig. 1B). It integrates over 1587 tools across 11 species. The related studies encompass three main areas of biological research, including cancer biology, developmental biology, and immunology. Second, the common single-cell data analysis procedure summarized from hundreds of publications can help researchers become quickly familiarized with the workflow and related steps. The tool parameters and usage scenarios extracted from publications can help users select appropriate analysis tools as well as specify optimal parameters for their own data processing. Third, the statistics based on the curated tools may help users track recent trends in methods development and further promote the design of new tools. Fourth, to facilitate the comparison of many tools, the min–max scaling method is used to normalize the citations of publications. Finally, the citation data can be automatically updated to keep the information up to date. The resource website will be updated periodically as new tools or articles become available. Furthermore, users can submit new tools or updates through the resource website directly.

The data extracted from hundreds of publications uncovered several notable trends in single-cell based research. In recent years, increasing studies utilized the 10X Genomics platform to perform single-cell sequencing as this technology enables time- and cost-effective sequencing of a large number of cells. According to our analysis, there is a trend that the single-cell technology will seek to harness a multi-omics approach by integrating genetics, epigenetics, transcriptomics, or proteomics in the future [12]. Furthermore, the development of single-cell and spatial transcriptome co-analysis has been very rapid. One of the representative tools that is used to perform such kinds of tasks is SNARE-seq [37] and MERFISH [38]. With regard to the development of tools with multi-functions, many software, including Millefy [39], HoneyBADGER [40], and landSCENT [41], process more than two steps in the analysis pipeline. This suggests that single-cell analysis tools tend to be integrated into a single analysis pipeline or multifunctional tools. The integration of these tools facilitates the design of user-friendly interfaces and greatly simplifies the analysis process. Furthermore, various single-cell multi-omics and spatial approaches will appear in the foreseeable future that will enable researchers to elucidate physiological and pathological processes at the single-cell level. Finally, more novel tools will be developed to meet the needs of multi-omics and spatial data analysis.

Since there are many studies on single-cell transcriptomes, one of the limitations is that our research is mainly focused on single-cell transcriptomes, the other omics analysis workflows remain to be added to the database. Moreover, with the development of single cell technology, there are more and more tools for single-cell analysis, and there may be some that we have overlooked. Single-cell proteomics is an emerging field that still faces many challenges [42]. In the future, we will focus on other single cell omics analysis processes, such as single-cell proteomics [43], scATAC [44], etc., and add them to the database timely. At the same time, we will also use our own analysis process to benchmark tools and recommend the use of tools.

The ultra-high resolution of single-cell sequencing provides new perspectives and opens new frontiers for researchers to understand many areas of biological sciences. The current hotspots of single-cell research focus on tumor heterogeneity, developmental phylogenies, and immunology. In the future, these research fields are expected to remain the major application areas of single-cell sequencing. We believe that SingleScan will substantially contribute to these emerging themes that scientists are only beginning to understand.

### Supplementary Information

**Additional file 1:** Detailed description of all the functional modules and manual for SingleScan database.

## Data Availability

All data are freely available at: http://cailab.labshare.cn/SingleScan

## References

[1] Grosselin K, Durand A, Marsolier J, Poitou A, Marangoni E, Nemati F, Dahmani A, Lameiras S, Reyal F, Frenoy O (2019). High-throughput single-cell ChIP-seq identifies heterogeneity of chromatin states in breast cancer. Nat Genet.

[2] Rotem A, Ram O, Shoresh N, Sperling RA, Goren A, Weitz DA, Bernstein BE (2015). Single-cell ChIP-seq reveals cell subpopulations defined by chromatin state. Nat Biotechnol.

[3] Ai S, Xiong H, Li CC, Luo Y, Shi Q, Liu Y, Yu X, Li C, He A (2019). Profiling chromatin states using single-cell itChIP-seq. Nat Cell Biol.

[4] Ku WL, Nakamura K, Gao W, Cui K, Hu G, Tang Q, Ni B, Zhao K (2019). Single-cell chromatin immunocleavage sequencing (scChIC-seq) to profile histone modification. Nat Methods.

[5] Kaya-Okur HS, Wu SJ, Codomo CA, Pledger ES, Bryson TD, Henikoff JG, Ahmad K, Henikoff S (2019). CUT&Tag for efficient epigenomic profiling of small samples and single cells. Nat Commun.

[6] Wang Q, Xiong H, Ai S, Yu X, Liu Y, Zhang J, He A (2019). CoBATCH for High-Throughput Single-Cell Epigenomic Profiling. Mol Cell.

[7] Buenrostro JD, Wu B, Litzenburger UM, Ruff D, Gonzales ML, Snyder MP, Chang HY, Greenleaf WJ (2015). Single-cell chromatin accessibility reveals principles of regulatory variation. Nature.

[8] Cusanovich DA, Daza R, Adey A, Pliner HA, Christiansen L, Gunderson KL, Steemers FJ, Trapnell C, Shendure J (2015). Multiplex single cell profiling of chromatin accessibility by combinatorial cellular indexing. Science.

[9] Moffitt JR, Lundberg E, Heyn H (2022). The emerging landscape of spatial profiling technologies. Nat Rev Genet.

[10] Vandereyken K, Sifrim A, Thienpont B, Voet T (2023). Methods and applications for single-cell and spatial multi-omics. Nat Rev Genet.

[11] Ren X, Wen W, Fan X, Hou W, Su B, Cai P, Li J, Liu Y, Tang F, Zhang F (2021). COVID-19 immune features revealed by a large-scale single-cell transcriptome atlas. Cell.

[12] Heumos L, Schaar AC, Lance C, Litinetskaya A, Drost F, Zappia L, Lücken MD, Strobl DC, Henao J, Curion F (2023). Best practices for single-cell analysis across modalities. Nat Rev Genet.

[13] Amezquita RA, Lun ATL, Becht E, Carey VJ, Carpp LN, Geistlinger L, Marini F, Rue-Albrecht K, Risso D, Soneson C (2020). Orchestrating single-cell analysis with Bioconductor. Nat Methods.

[14] Hafemeister C, Satija R (2019). Normalization and variance stabilization of single-cell RNA-seq data using regularized negative binomial regression. Genome Biol.

[15] Satija R, Farrell JA, Gennert D, Schier AF, Regev A (2015). Spatial reconstruction of single-cell gene expression data. Nat Biotechnol.

[16] Hao Y, Hao S, Andersen-Nissen E, Mauck WM, Zheng S, Butler A, Lee MJ, Wilk AJ, Darby C, Zager M (2021). Integrated analysis of multimodal single-cell data. Cell.

[17] Stuart T, Butler A, Hoffman P, Hafemeister C, Papalexi E, Mauck WM, Hao Y, Stoeckius M, Smibert P, Satija R (2019). Comprehensive Integration of Single-Cell Data. Cell.

[18] Butler A, Hoffman P, Smibert P, Papalexi E, Satija R (2018). Integrating single-cell transcriptomic data across different conditions, technologies, and species. Nat Biotechnol.

[19] Wolf FA, Angerer P, Theis FJ (2018). SCANPY: large-scale single-cell gene expression data analysis. Genome Biol.

[20] Yuan H, Yan M, Zhang G, Liu W, Deng C, Liao G, Xu L, Luo T, Yan H, Long Z (2019). CancerSEA: a cancer single-cell state atlas. Nucl Acids Res.

[21] Cao Y, Zhu J, Han G, Jia P, Zhao Z. scRNASeqDB: a database for gene expression profiling in human single cell by RNA-seq. bioRxiv 2017:104810. 10.3390/genes8120368 PMC5748686 29206167

[22] Franzén O, Gan LM, Björkegren JLM: PanglaoDB: a web server for exploration of mouse and human single-cell RNA sequencing data. Database (Oxford) 2019, 2019. 10.1093/database/baz046 PMC6450036 30951143

[23] Zappia L, Phipson B, Oshlack A (2018). Exploring the single-cell RNA-seq analysis landscape with the scRNA-tools database. PLoS Comput Biol.

[24] Zappia L, Theis FJ (2021). Over 1000 tools reveal trends in the single-cell RNA-seq analysis landscape. Genome Biol.

[25] Lee J, Hyeon DY, Hwang D (2020). Single-cell multiomics: Technologies and data analysis methods. Exp Mol Med.

[26] Stoeckius M, Hafemeister C, Stephenson W, Houck-Loomis B, Chattopadhyay PK, Swerdlow H, Satija R, Smibert P (2017). Simultaneous epitope and transcriptome measurement in single cells. Nat Methods.

[27] Hou Y, Guo H, Cao C, Li X, Hu B, Zhu P, Wu X, Wen L, Tang F, Huang Y (2016). Single-cell triple omics sequencing reveals genetic, epigenetic, and transcriptomic heterogeneity in hepatocellular carcinomas. Cell Res.

[28] Luecken MD, Theis FJ (2019). Current best practices in single-cell RNA-seq analysis: A tutorial. Mol Syst Biol.

[29] Trapnell C, Cacchiarelli D, Grimsby J, Pokharel P, Li S, Morse M, Lennon NJ, Livak KJ, Mikkelsen TS, Rinn JL (2014). The dynamics and regulators of cell fate decisions are revealed by pseudotemporal ordering of single cells. Nat Biotechnol.

[30] Qiu X, Mao Q, Tang Y, Wang L, Chawla R, Pliner HA, Trapnell C (2017). Reversed graph embedding resolves complex single-cell trajectories. Nat Methods.

[31] Qiu X, Hill A, Packer J, Lin D, Ma YA, Trapnell C (2017). Single-cell mRNA quantification and differential analysis with Census. Nat Methods.

[32] Wolf FA, Hamey FK, Plass M, Solana J, Dahlin JS, Göttgens B, Rajewsky N, Simon L, Theis FJ (2019). PAGA: graph abstraction reconciles clustering with trajectory inference through a topology preserving map of single cells. Genome Biol.

[33] Li H, Durbin R (2009). Fast and accurate short read alignment with Burrows-Wheeler transform. Bioinformatics.

[34] Robinson MD, McCarthy DJ, Smyth GK (2010). edgeR: a Bioconductor package for differential expression analysis of digital gene expression data. Bioinformatics.

[35] Langmead B, Wilks C, Antonescu V, Charles R (2019). Scaling read aligners to hundreds of threads on general-purpose processors. Bioinformatics.

[36] Zheng GX, Terry JM, Belgrader P, Ryvkin P, Bent ZW, Wilson R, Ziraldo SB, Wheeler TD, McDermott GP, Zhu J (2017). Massively parallel digital transcriptional profiling of single cells. Nat Commun.

[37] Chen S, Lake BB, Zhang K (2019). High-throughput sequencing of the transcriptome and chromatin accessibility in the same cell. Nat Biotechnol.

[38] Fang R, Xia C, Close JL, Zhang M, He J, Huang Z, Halpern AR, Long B, Miller JA, Lein ES (2022). Conservation and divergence of cortical cell organization in human and mouse revealed by MERFISH. Science.

[39] Ozaki H, Hayashi T, Umeda M, Nikaido I (2020). Millefy: visualizing cell-to-cell heterogeneity in read coverage of single-cell RNA sequencing datasets. BMC Genomics.

[40] Fan J, Lee HO, Lee S, Ryu DE, Lee S, Xue C, Kim SJ, Kim K, Barkas N, Park PJ (2018). Linking transcriptional and genetic tumor heterogeneity through allele analysis of single-cell RNA-seq data. Genome Res.

[41] Chen W, Morabito SJ, Kessenbrock K, Enver T, Meyer KB, Teschendorff AE (2019). Single-cell landscape in mammary epithelium reveals bipotent-like cells associated with breast cancer risk and outcome. Commun Biol.

[42] Redit C, Cha S, Ai N (2023). Single-cell proteomics: challenges and prospects. Nat Methods.

[43] Schoof EM, Furtwängler B, Üresin N, Rapin N, Savickas S, Gentil C, Lechman E, Keller UAD, Dick JE, Porse BT (2021). Quantitative single-cell proteomics as a tool to characterize cellular hierarchies. Nat Commun.

[44] Buenrostro JD, Corces MR, Lareau CA, Wu B, Schep AN, Aryee MJ, Majeti R, Chang HY, Greenleaf WJ (2018). Integrated single-cell analysis maps the continuous regulatory landscape of human hematopoietic differentiation. Cell.

