# Supplementary material for: SingleScan: a comprehensive resource for single-cell sequencing data processing and mining
Source: BMC Bioinformatics. 2023 Dec 7;24:463. doi: 10.1186/s12859-023-05590-9 (PMC10704760; doi:10.1186/s12859-023-05590-9)
Supplement: Supplementary file 1 — Additional file 1: Detailed description of all the functional modules and manual for SingleScan database. [file 12859_2023_5590_MOESM1_ESM.docx]

Supplementary Materials for

**SingleScan: a comprehensive resource for single-cell sequencing data processing and mining**

Kun Wang^1,#^, Xiao Zhang^2,3,#^, Hansen Cheng^1^, Wenhao Ma^1^,Guangchao Bao^1^, Liting Dong^1^, Yixiong Gou^1^, Jian Yang^1,*^, Haoyang Cai^1,*^

**
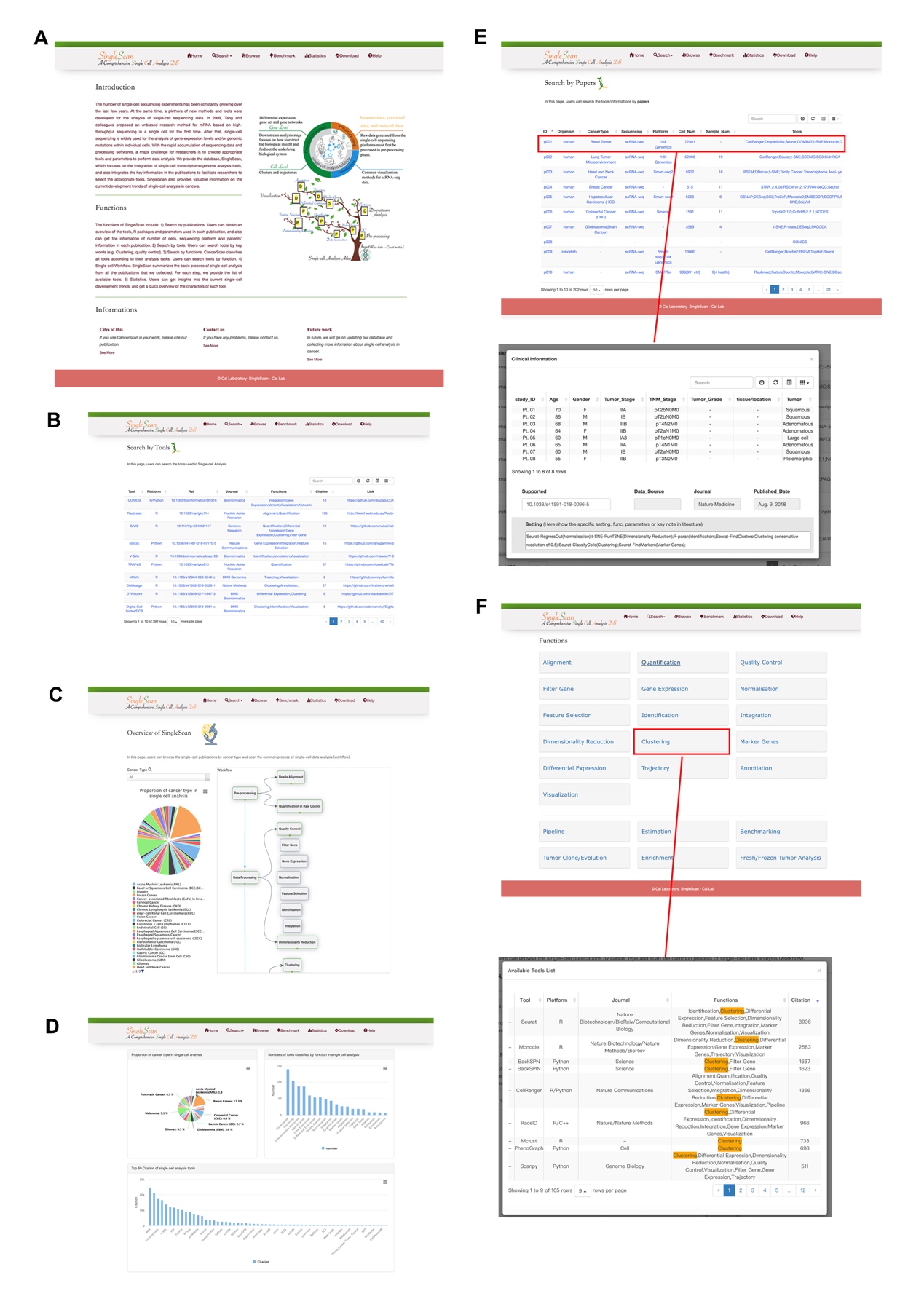
**

**Figure S1. The main interface of SingleScan.** (A) The ‘Home’ page of SingleScan. (B)’Search by Tools’ page shows the quick view of tools. (C) The ‘Browser’ page shows the pipelines of single-cell data analysis. (D) Statistical analysis. (E) The ‘Search by paper’ page shows the details of each research, including sample number, clinical information, etc. (F) The ‘Search by function’ page shows the tools of each functional module.


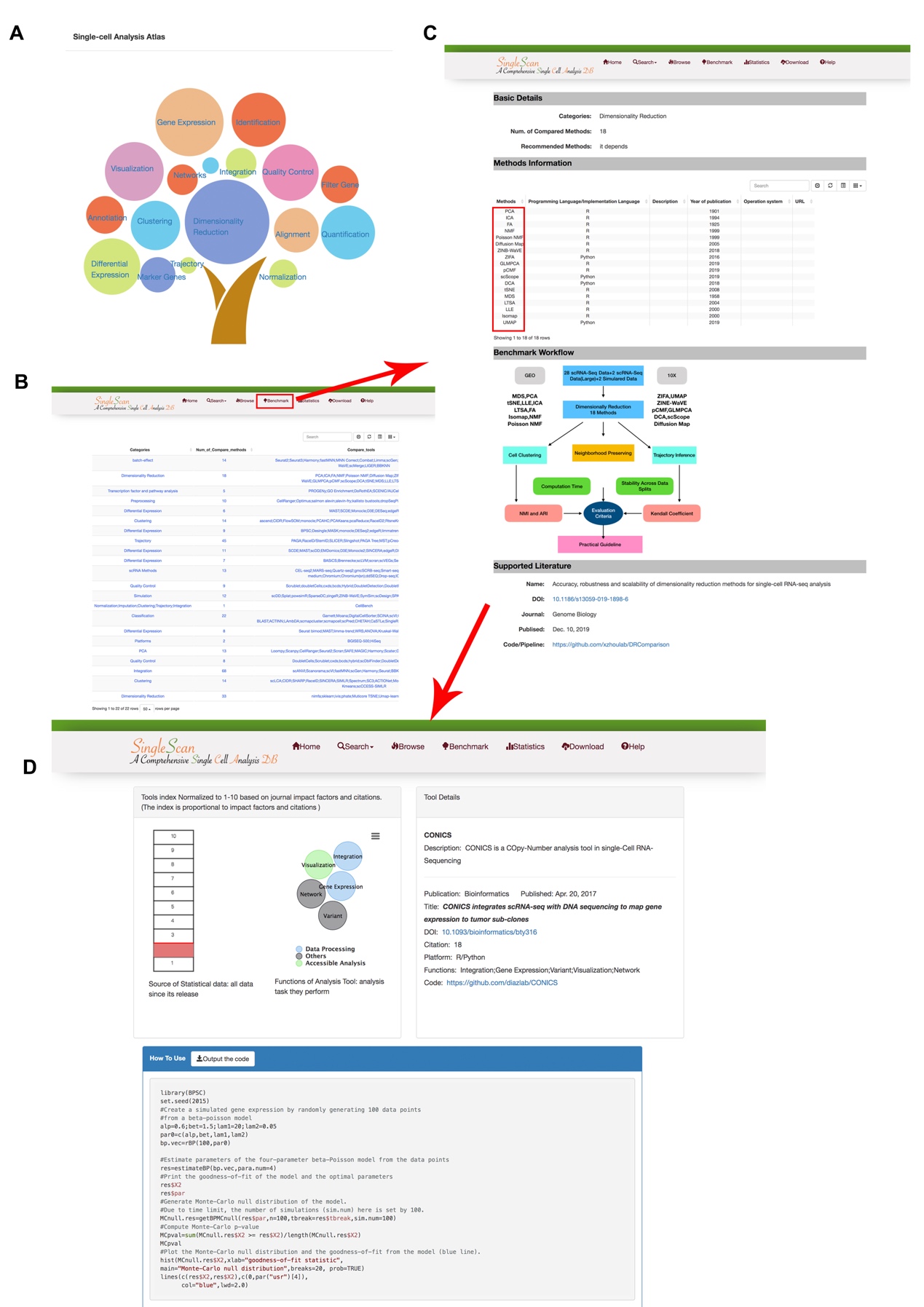


**Figure S2. Details of a functional module and its associated benchmark repertoires.** (A) single-cell tools with different modules. (B) A quick view of ‘benchmark’ page. (C) A benchmark of single-cell methods for each tool category. (D) Detailed information about a tool.


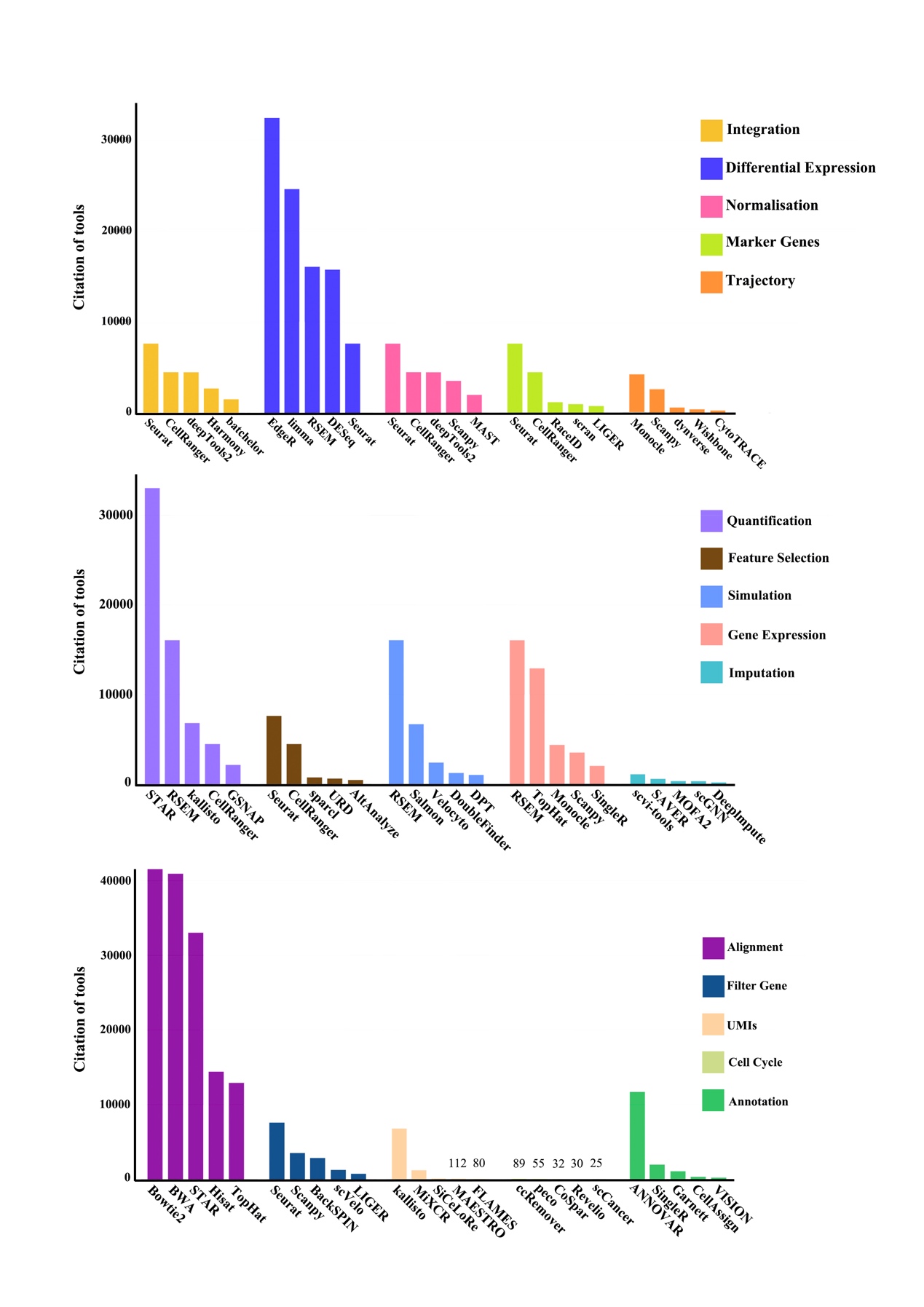


**Figure S3. Tools are sorted by citations separately within each functional group** (the remaining 15 functions in **Figure 3C**).


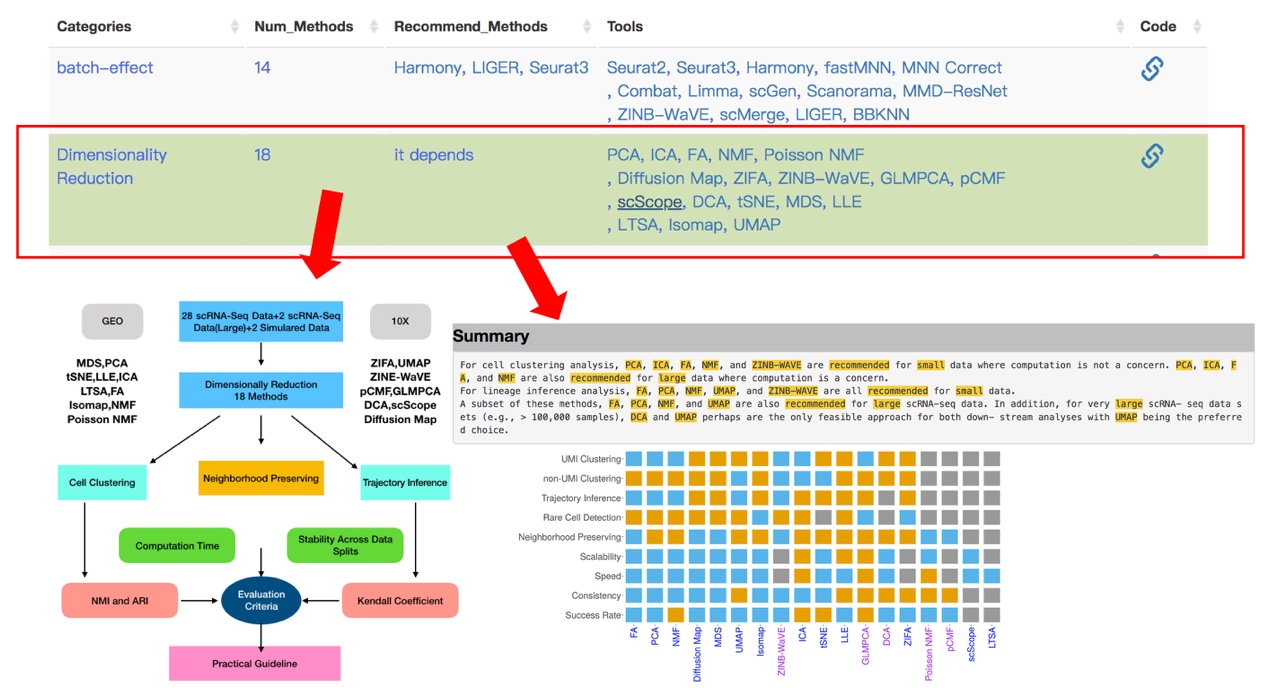


**Figure S4. An example of ‘Benchmark’ page.**

**
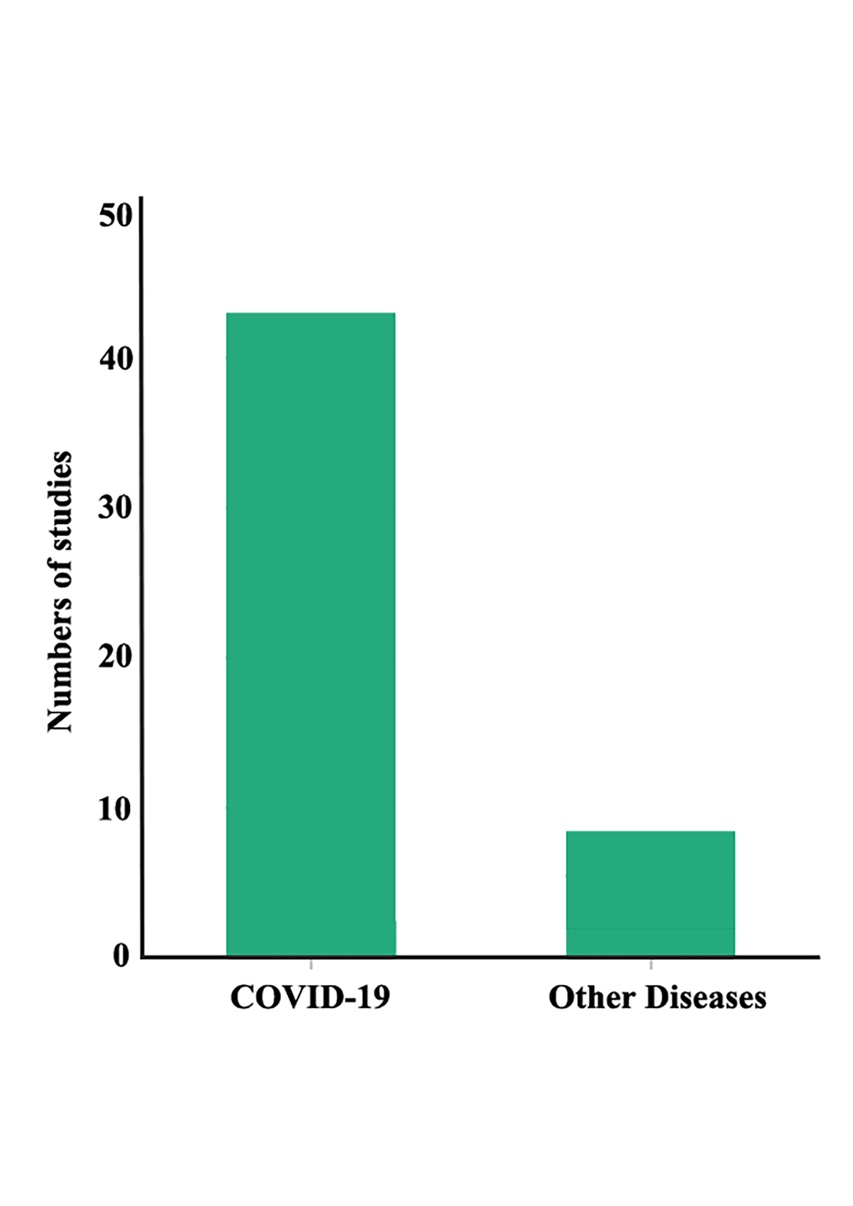
**

**Figure S5. Number of publications on disease.**

**Table S1. The description of function modules**

| **Functions** | **Description** |
| --- | --- |
| Alignment | Genome alignment, align reads to a reference genome |
| UMIs | Processing Unique Molecular Identifiers (UMI) sequence |
| Quantification | Quantification from reads to gene expression (matrix) |
| Quality Control | Remove low-quality cells based on setting conditions |
| cell cycle | Remove the impact of cycling cells on analysis |
| Filter Gene | Remove lowly-expressed genes |
| Gene Expression | Inferring gene expression programs from single-cell sequencing data |
| Normalisation | Normalization addresses unwanted variation by e.g. scaling count data to obtain correct relative gene expression abundances between cells |
| Feature Selection | Filtered to keep only genes that are “informative” of the variability in data. Highly variable genes (HVGs) are often used |
| Identification | Identify cell types according to a reference dataset |
| Integration | Integration of single-cell sequencing datasets with other single-cell data |
| Networks | Co-regulated gene networks analysis |
| Dimensionality Reduction | Project cells from high-dimensional space to low-dimensional space |
| Clustering | Cell grouping based on expression profiles |
| Marker Genes | Calculate the representative genes of each cell-type after clustering |
| Differential Expression | Test for differential expression across cell-types |
| Trajectory | Trajectory analysis the data are regarded as a snapshot of a dynamic process |
| Imputation | Estimation of expression observed zeros in the gene-cell expression matrix of the single-cell sequencing datasets |
| Annotation | Automatically annotate the cell type according to some database |
| Visualization | Visualizing the results of single-cell sequencing data analysis |
